# Demographic variation and socioeconomic inequalities in all forms of malnutrition among children aged 6 months to 9 years: findings from the Vietnamese General Nutrition Survey 2020

**DOI:** 10.1136/bmjph-2024-001177

**Published:** 2025-02-04

**Authors:** Pui Yee Tan, Somphos Vicheth Som, Son Duy Nguyen, Xiaomian Tan, Do Tranh Tran, Nga Thuy Tran, Van Khanh Tran, Louise Dye, J Bernadette Moore, Samantha Caton, Hannah Ensaff, Xiaodong Lin, Geoffry Smith, Yun Yun Gong

**Affiliations:** 1 School of Food Science and Nutrition, Faculty of Environment, University of Leeds, Leeds, UK; 2 Section of International Health, Department of Health Sciences, Vrije Universiteit Amsterdam, Amsterdam, The Netherlands; 3 Division of Human Nutrition and Health, Wageningen University and Research, Wageningen University and Research, Wageningen, The Netherlands; 4 Nutrition Surveillance and Policy Department, National Institution of Nutrition, Hanoi, Vietnam; 5 Department of Micronutrient, National Institution of Nutrition, Hanoi, Vietnam; 6 Institute for Sustainable Food and Department of Psychology, The University of Sheffield, Sheffield, UK; 7 Sheffield Centre for Health and Related Research (SCHARR), School of Medicine and Population Health, University of Sheffield, Sheffield, UK; 8 Global Sustainable Development, University of Warwick, Coventry, UK; 9 International Life Sciences Institute (ILSI) Southeast Asia Region, Singapore

**Keywords:** public health, cross-sectional studies, epidemiology, nutrition surveys, epidemiologic factors

## Abstract

**Introduction:**

The double burden of malnutrition (DBM) is a global public health challenge. This study examined the prevalence of population-level DBM, its demographic and socioeconomic determinants as well as the associations between anthropometric indicators of undernutrition and overnutrition, and micronutrient deficiencies (MNDs), among Vietnamese children, using the data from the nationally representative General Nutrition Survey 2020.

**Methods:**

Data on anthropometric parameters, micronutrient biomarkers, demographic and socioeconomic indicators for 7289 children aged 6 months to 9 years were analysed. Determinants of malnutrition were assessed using logistic regressions, reported as OR with 95% CIs.

**Results:**

At national level, 12.7%, 10.5% and 4.7% of children were stunted, underweight and wasted/thin, while 7.3% and 7.1% were overweight and obese, respectively. Low serum zinc, anaemia and iron deficiency were the common MNDs observed, affecting 53.1%, 15.2% and 13.9% of the study participants. Older children aged 2–4 years (OR (95% CI) 1.43 (1.20, 1.72)), ethnic minorities (5.94 (3.78, 9.36)) and those living in mountainous areas (5.06 (1.18, 14.42)) had increased odds of stunting, whereas reduced odds were found in children from the richest quintile (0.13 (0.05, 0.32)). Similar determinants were associated with underweight and MNDs. Males (1.43 (1.16, 1.76)), children aged 5–9 years (10.02 (6.71, 14.97) and children from the richest quintile (2.91 (1.20, 7.05)) had increased odds of overweight. Children with anaemia, low serum retinol and low serum zinc had increased odds of stunting and underweight than non-micronutrient-deficient children (adjusted OR=1.43–1.71). Compared with children without MNDs, those with ≥3 MNDs had almost double the odds of stunting and underweight, whereas those with ≤3 MNDs had reduced odds of overweight (adjusted OR=0.38–0.60).

**Conclusions:**

Significant demographic variation and socioeconomic inequalities in child malnutrition were identified. National policies and programmes in Vietnam should address age-specific, sex-specific, geographical and socioeconomic disparities to accelerate progress in reducing child malnutrition.

**WHAT IS ALREADY KNOWN ON THIS TOPIC**

- The double burden of malnutrition (DBM) refers to the co-existence of undernutrition including micronutrient deficiencies (MNDs), and overnutrition.
- Although DBM is a major public health issue that affects every country worldwide, it is particularly pervasive in low- and middle-income countries.
- There is no recent data investigating the magnitude of the DBM in Vietnam and how prevalence of DBM differs across different demographic and socioeconomic groups, especially among infants and young children.

**WHAT THIS STUDY ADDS**

- Our analysis of the Vietnamese national representative General Nutrition Survey (GNS) 2020 data demonstrated the extent of the co-existence of each aspect of the DBM among children in Vietnam, and demographic variation and socioeconomic inequalities in child malnutrition were identified.
- Although child stunting reduction is on track to meet the target of 40% reduction by 2025, as set by the Global Nutrition Targets, the prevalence remains high at 10%−15% in 2020.
- Meanwhile, overweight and obesity rates in school-aged children are considerably off track to meet the target, with the prevalence in urban areas increasing from 8.5% in 2010 to 31% in 2020.
- Stunting, underweight and MNDs were more prevalent in infants and young children, particularly in ethnic minorities, living in the rural and mountainous areas, and in poorer families.
- Childhood overweight and obesity were particularly evident among school-aged children, males, those living in urban areas and those from wealthier families.
- Low serum zinc (53%), anaemia (15%) and iron deficiency (14%) were the most common MNDs, and prevalence of low serum retinol was relatively low (<7%) in this population.

**HOW THIS STUDY MIGHT AFFECT RESEARCH, PRACTICE OR POLICY**

- These findings emphasise the need for double-duty actions to simultaneously address all forms of child malnutrition in Vietnam.
- National food and nutrition policies and programmes in Vietnam should address age-specific, sex-specific, geographical and socioeconomic disparities to ensure equitable access and opportunities for all, to accelerate progress in reducing child malnutrition.

## Introduction

 The double burden of malnutrition (DBM) currently affects every country worldwide.^1^ It is characterised by the co-existence of undernutrition including micronutrient deficiencies (MNDs) or ‘hidden hunger’, and overnutrition, manifesting at the individual, household or population level.^2^ Child malnutrition significantly impacts national economies by reducing human productivity and straining healthcare systems.^3^ Undernutrition is particularly detrimental for children under 5 years, heightening the risk of child mortality and morbidity, while the associated deficiencies in iron, zinc, folate, iodine and vitamin A increase susceptibility to infections, impair immune function and hinder growth and development.^4^ Children living with overweight and obesity are more likely to become adults with obesity, facing elevated risks of obesity-related non-communicable diseases (NCDs) at a younger age.^2 5^ Moreover, life course evidence shows that early exposure to undernutrition can predispose individuals to later-life overweight and obesity, further increasing the risk of NCDs.^6^ Thus, childhood is a critical period for addressing the burden of nutrition-related diseases and preventing intergenerational malnutrition and its long-term consequences.

Vietnam has committed to achieving the United Nations Sustainable Development Goals (SDGs), particularly SDG 2.2, which calls for an end to all forms of malnutrition.^7^ By 2030, the Vietnamese government aims to reduce stunting and underweight in children aged under 5 years to below 20% and 10%, respectively, and to reduce the prevalence of overweight in urban areas to below 10%.^7^ The General Nutrition Survey (GNS) reported substantial reductions in stunting among Vietnamese children aged under 5 years, from 43.3% in 2000 to 29.3% in 2010, and to 24.5% in 2015, and in underweight from 30.1% in 2000 to 17.5% in 2010, and 13.9% in 2015.^7^,^9^ The South-East Asian Nutrition Surveys II 2020 indicated clear disparities in malnutrition across age, sex and regions among Vietnamese children aged 0.5–11.9 years.^10^ Stunting was particularly prevalent in children aged 1–3.9 years (12.1%; overall 7.6%), while underweight was higher among boys aged 4–6.9 years in rural areas (14.8%; overall 5.9%).^10^ Conversely, wasting was more common among girls of the same age in urban areas (17.7%; overall 3.8%). Prevalence of overweight and obesity increased with age, with 32.7% of primary school children aged 7–11.9 years affected, particularly boys in urban areas (56.7%; overall 23.4%).

Vietnam’s ethnic diversity further complicates the malnutrition challenge. The Kinh ethnic group constitutes 85.3% of the population, while the remaining 14.7% comprises 53 ethnic minority groups.^11^ These ethnic minorities, particularly those in rural and mountainous areas of the Northern and Central Highlands regions, face higher rates of undernutrition compared with the Kinh majority in urban areas.^12^ Meanwhile, urban regions are witnessing a growing trend of childhood obesity. These regional and ethnic disparities, rooted in socioeconomic inequalities, pose significant challenges for Vietnam to achieve its national goals in ending all forms of malnutrition.^13^

MNDs have been under-recognised, despite their negative impact on health.^14^ MNDs are increasingly evident in individuals with both undernutrition and overnutrition due to their nutrient-poor, energy-dense or energy-inadequate diets,^15 16^ further complicating the malnutrition landscape.^17^ A pooled analyses of national surveys spanning 2003–2019 by Stevens *et al* reported that nearly 66% of children under 5 years were suffering from at least one of the three common MNDs (iron, zinc and vitamin A), with 56%, 19%, 14% and 6% experiencing iron, zinc, vitamin A and vitamin D deficiencies, respectively.^18^ The SEANUTs II study showed similar prevalences of vitamin A deficiency (6.2%) and zinc deficiency (60.8%) in Vietnamese children aged 4–11.9 years, while a higher prevalence of vitamin D deficiency (31.1%) and a lower prevalence of iron deficiency (ID) (3.7%) were observed.^10^

To date, there is limited recent data on the prevalence and associations between multiple burdens of malnutrition, and relevant demographic and socioeconomic factors in Vietnam, especially among children. Therefore, this study aimed to determine the prevalence of population-level of DBM including undernutrition (stunting, underweight and wasting/thinness), overnutrition (overweight and obesity) and MNDs (anaemia, iron, vitamin A or retinol and zinc deficiencies), in Vietnamese children aged 6 months to 9 years. Furthermore, the study explored the demographic and socioeconomic determinants of DBM, and examined the relationships between MNDs and anthropometric indicators of undernutrition and overnutrition, using data from the nationally representative Vietnamese GNS 2020.

## Methods

### Study population and study design

Secondary analysis of the nationally representative Vietnamese GNS 2020 was performed. The GNS is conducted every 10 years by the National Institute of Nutrition (NIN). A multistage cluster sampling technique was used to select a representative sample of Vietnamese population and avoid bias. Clusters corresponded to census enumeration areas (EAs) and were treated as primary sampling units. The first stage of sampling involved selection of a subset of provinces across six geographical areas in Vietnam, namely (i) Northern midlands and mountainous areas, (ii) Central Highlands, (iii) Red River Delta (including Hanoi Capital); (iv) North-central and central coastal areas, (v) Southeast (including Ho Chi Minh City) and (vi) Mekong River Delta. EAs were selected from 25 out of 63 provinces to represent the six geographical areas. In the second stage of sampling, the selected EAs were allocated to urban, rural and mountainous strata to ensure adequate representation of urbanicity. In the final stage of sampling, eligible individuals from each EAs were randomly selected for enrolment in GNS 2020.

Participants encompassed various subgroups, including those aged 0–5, 6–9, 10–14 and 15–49 years, along with pregnant and lactating women. For children aged ≤9 years, informed written consent was obtained from their parents/caregivers. Individuals who declined to respond or did not provide written informed consent were excluded from the survey. A total of 20 864 individuals were enrolled in GNS 2020. In this study, individuals were excluded if they had missing age data or if they aged <6 months and >9 years. Infants aged 0–5 months were excluded from the analysis due to the small sample size, particularly for micronutrient data, which may introduce bias. Additionally, the prevalence of malnutrition among adolescents (aged 10–18 years) was reported in our previous publication,^19^ thus this age group was removed to avoid redundancy. Finally, a total of 7829 children aged 6 months to 9 years who had complete demographic and socioeconomic indicators were included for analysis.

### Anthropometric indicators of malnutrition

Anthropometric measurements including body weight and height/length were taken in children wearing light clothes and without shoes. Measurements were taken twice, and the mean was calculated. The anthropometric status of the children was classified according to the WHO Child Growth Standards for children under 5 years,^20^ and WHO Growth Reference for children aged 5–9 years.^21^ Stunting was defined as height-for-age z-scores (HAZ) <−2 SD. Underweight was defined as weight-for-age z-scores (WAZ) <−2 SD. For children under 5 years, wasting was defined as weight-for-height z-scores (WHZ) <−2 SD, whereas for children aged 5–9 years, thinness was defined as body mass index (BMI)-for-age z-scores (BAZ) <−2 SD. BMI was defined as weight (kg) divided by the square of height (m). Overweight and obesity were defined as WHZ >+2 SD to ≤+3 SD and >+3 SD for children under 5 years,^20^ and BAZ >+1 SD to ≤+2 SD and >+2 SD for children aged 5–9 years,^21^ respectively. Children with HAZ and WHZ <−6 SD and >+6 SD or WAZ <−6 SD or >+5 SD were flagged as outliers and were removed as per WHO recommendation.^20 21^

### Biochemical analysis and definition of micronutrient deficiencies

The selection of micronutrients for this study was based on three criteria: (1) availability of biomarkers in the GNS 2020 dataset, (2) prevalence and severity of deficiencies in Vietnam and (3) alignment with government priorities as outlined in the National Nutrition Strategy 2025 and 2030. As a result, three micronutrients including, iron, retinol and zinc were selected. Additionally, anaemia was considered as an outcome related to ID, and was included among the MNDs. A total of 5 mL non-fasting blood was collected between 07:00 and 09:00 hours from each participant. Haemoglobin (Hb) was measured immediately after blood sample collection using a HemoCue Hb 301 analyser (HemoCue, Angholm, Sweden). Any participant who had severe anaemia (<7.0 g/L for children) was referred to the physician at the Commune Health Centre.^22^

Serum ferritin, transferrin receptor (sTfR), retinol binding protein, C reactive protein (CRP) and α-1-acid glycoprotein (AGP) concentrations were measured by VitMin Lab ELISAassays (VitMin Lab, Willstaett, Germany). Accuracy was tested by controls from the Centers for Disease Control and Prevention (CDC), USA and National Institute of Biological Standards and Control. Serum retinol concentration was determined by reverse-phase liquid chromatography with tandem mass spectrometry (3000 Otrap, Sciex, Framingham, USA) with quality controls approved by CDC, USA. Serum zinc concentration was analysed by flame atomic absorption spectrophotometer (GBC, Avanta+, Keysborough, Australia) using trace element-free procedures, and powder-free gloves, and results were verified using reference materials (Liquicheck, Bio-Rad Laboratories, California, USA). The within-assay coefficient of variation for serum ferritin, zinc, retinol, CRP and AGP ranged from 2.9% to 7.1%, and between-assay variability was <10% for all the biomarkers.

Anaemia was defined as Hb concentrations <110 g/L and<115 g/L for children under 5 and 5–9 years, respectively.^22^ Different cut-offs were applied to define ID (ID) in children of different age and inflammatory status. ID was defined as serum ferritin concentrations <12 µg/L (under 5 years) and <15 µg/L (5–9 years) in children without inflammation, whereas, cut-offs of <30 µg/L (under 5 years) and <70 µg/L (5–9 years) were applied for children with inflammation. Inflammation was defined as CRP >5 mg/L and/or AGP >1 g/L.^23^ Iron deficiency anaemia (IDA) was defined as the presence of both anaemia and ID. Low serum retinol was defined as serum retinol concentrations <0.70 µmol/L.^24^ Low serum zinc was defined as serum zinc concentration <9.9 µmol/L (morning, non-fasting) or <8.7 µmol/L (afternoon, non-fasting) according to International Zinc Nutrition Consultative Group.^25^ Total number of MNDs included anaemia, ID, low serum retinol and low serum zinc.

### Demographic and socioeconomic indicators

A structured questionnaire was used to collect demographic and socioeconomic data from the parents/caregivers including child’s age, sex, ethnicity (Kinh and other ethnicities), geographical areas and area of residence (urban, rural and mountainous). Household wealth index was determined according to the Demographic and Health Survey (DHS) Wealth Index.^26^ It was computed based on household ownership of selected assets, materials used for housing construction, types of water access and sanitation facilities by the statisticians from NIN. The household wealth index was grouped into quintiles for further analysis: poorest (Q1), poorer (Q2), middle (Q3), richer (Q4) and richest (Q5) quintiles. The household wealth index was used as a proxy indicator for children’s socioeconomic status.

### Statistical analysis

All statistical analyses were conducted using STATA (V.18; StataCorp, Cary, North Carolina, USA). All analyses were weighted and accounted for complex survey design using svyset commands for individual sampling weight, clustering and stratification. Baseline characteristics by area of residence were reported as mean±SD for continuous variables and number (percentage) for categorical variables. These were compared using one-way between-groups analysis of variance with Bonferroni post hoc tests for continuous variables, and the χ^2^ test of association for categorical variables. Univariate logistic regressions assessed associations between demographic and socioeconomic factors and all forms of malnutrition. Both univariate and multivariate logistic regressions evaluated associations between undernutrition or overnutrition and MNDs, adjusting for significant covariates including age, sex, area of residence, wealth index and inflammation. Ethnicity and geographical area were not included due to multicollinearity. Crude OR or adjusted OR (AOR) with respective 95% CIs were reported. P value <0.05 was considered statistically significant. Sensitivity analyses were conducted in two ways: (i) by comparing randomly selected subsamples (representing 50% of the total population) within the overall samples and (ii) by testing two logistic regression models, with and without adjustments.

## Result

General characteristics, anthropometric parameters and micronutrient biomarkers of the study participants from rural, urban and mountainous areas are reported in table 1. See online supplemental table S1 for the number of participants with missing data for each variable. The average age±SD of participants was 5±3 years, with 48.5% being females. A significantly larger proportion of the children in urban areas were from the richest quintile (Q5) compared with those from the mountainous areas (17.3% vs 1.6%; p=0.005). These children came mainly from the Kinh major ethnic group (93.5% vs 36.3%; p<0.001). All anthropometric parameters except for HAZ were significantly higher in children from urban areas, compared with rural and/or mountainous areas (p<0.05). With respect to micronutrient biomarkers, concentrations of serum zinc were significantly higher in children from urban areas, whereas serum transferrin receptor were significantly lower (p<0.05). See online supplemental table S2 for the mean (SD) age, anthropometric and biomarkers of micronutrients of the Vietnamese children by anthropometric indicators of malnutrition.

**Table 1:** General characteristics, anthropometric parameters and micronutrient biomarkers of the Vietnamese children, and by area of residence (n=7829)

Variables	Total (n=7829)	Urban (n=2540)	Rural (n=3840)	Mountainous (n=1449)	P value
Age (years)	5±3	5±3	5±3	5±3	0.330
Age group (%)					0.405
<2 years	16.0	14.6	16.8	16.0	
2–4 years	31.6	31.1	31.8	31.6	
5–9 years	52.4	54.3	51.4	52.4	
Sex (%)					0.433
Males	51.5	48.6	49.2	46.1	
Females	48.5	51.4	50.8	53.9	
Geographical area (%)					<0.001
Northern mountains	15.5	4.1	0	86.1	
Red River Delta	26.6	33.8	30.8	0	
North Central and Central Coastal	22.4	18.1	31.8	0	
Central Highlands	6.0	12.5	0	13.9	
Southeast	15.2	21.7	16.2	0	
Mekong River Delta	14.3	9.8	21.2	0	
Ethnicity (%)					<0.001
Kinh	85.0	93.5	95.4	36.3	
Others	15.0	6.5	4.6	63.7	
Wealth index quintiles (%)					0.005
Poorest (Q1)	13.6	5.5	8.3	45.6	
Poorer (Q2)	16.3	12.9	17.5	18.6	
Middle (Q3)	22.6	21.9	27.0	9.5	
Richer (Q4)	30.2	22.3	36.4	24.7	
Richest (Q5)	17.3	37.4	10.8	1.6	
Anthropometric parameters					
Weight (kg)	19.0±12.5	19.9±11.1a	18.9±9.1	17.3±21.6b	0.004
Height (cm)	105.5±20.1	10.6.9±20.9a	10.5.7±16.4a	102.0±19.2b	0.005
Height-for-age z-score	−0.65±1.28	−0.37±1.27a	−0.60±1.22b	−1.32±1.29c	<0.001
Weight-for-age z-score	−0.40±1.42	−0.14±1.44a	−0.35±1.40a	−1.04±1.24b	0.001
Weight-for-height z-score	−0.08±1.14	−0.06±1.17	−0.06±1.13	−0.22±1.06	0.125
Body mass index-for-age z-score	0.01±1.34	0.13±1.41a	0.02±1.34	−0.25±1.11b	0.016
Body mass index units	16.3±4.0	16.8±5.8a	16.2±2.9	15.3±3.5b	0.007
Blood biomarkers					
Haemoglobin (g/dL)	12.3±1.2	12.3±1.2	12.3±1.1	12.0±1.3	0.050
Serum ferritin (µg/L)	39.7±1.3	38.9±0.9	39.7±1.8	40.7±3.2	0.785
Serum transferrin receptor (mg/L)	6.2±2.6	6.0±2.4a	6.1±2.3a	6.7±3.3b	0.009
Body iron store	5.5±3.9	5.4±3.8	5.5±3.7	5.3±4.5	0.821
Serum zinc (µmol/L)	9.9±6.3	10.7±6.8a	10.0±6.4a	8.0±4.1b	0.003
Serum retinol (µmol/L)	1.2±0.4	1.1±0.4	1.1±0.3	1.0±0.3	0.089
Retinol-binding protein (µmol/L)	1.1±0.4	1.1±0.4	1.1±0.4	1.0±0.4	0.490
C reactive protein (mg/L)	1.6±4.5	1.8±4.9	1.5±4.0	1.6±5.0	0.375
α−1-acid glycoprotein (mg/L)	0.8±0.3	0.7±0.3	0.7±0.3	0.8±0.3	0.591

a,b,cContinuous variables with values not sharing a common lowercase letter are significantly different at p<0.05.

Continuous and categorical variables presented as mean±SD and percentage, respectively.

Serum ferritin concentration (µg/L) presented as geometric mean and SE due to non-normal distribution.

Differences in categorical and continuous variables between area of residence were performed using χ2 test of association and one-way between-groups analysis of variance with Bonferroni post hoc tests.

### Prevalence of different forms of malnutrition by area of residence and age group

**Figure 1:**
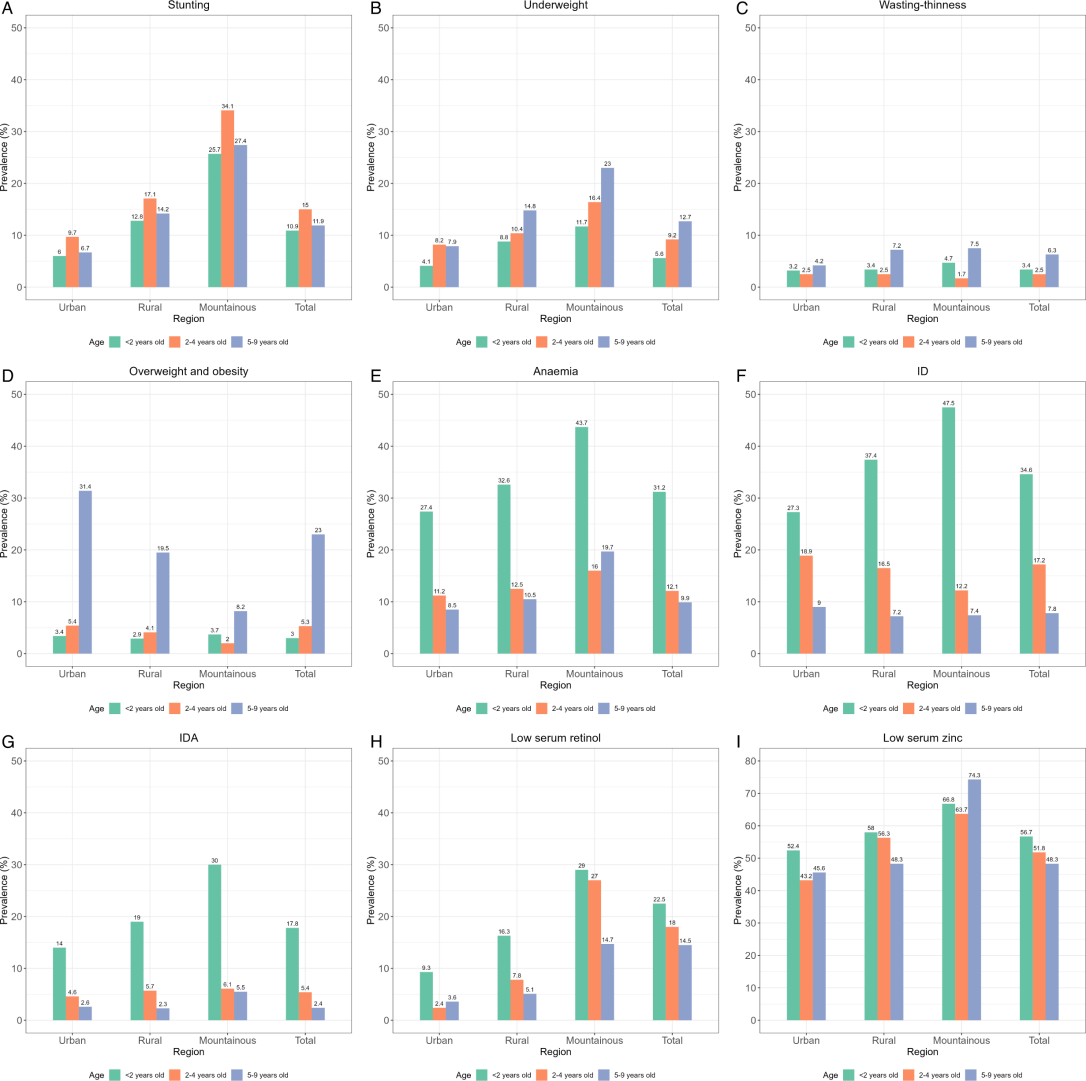
Prevalence of different forms of malnutrition by area of residence and age group among Vietnamese children. (**A**) Stunting. (**B**) Underweight. (**C**) Wasting/Thinness. (**D**) Overweight and obesity. (**E**) Anaemia. (**F**) Iron deficiency (ID). (**G**) Iron deficiency anaemia (IDA). (**H**) Low serum retinol. (**I**) Low serum zinc. Prevalence was estimated based on sampling weight.

In addition to the stratified prevalence shown in figure 1, the overall population-level prevalence of stunting, underweight and wasting/thinness was 12.7%, 10.5% and 4.7%, respectively. Furthermore, 7.3% and 7.1% were overweight and obese, respectively. Low serum zinc affected more than half of the Vietnamese children (53.1%). Prevalence of ID, anaemia, IDA and low serum retinol was 15.2%, 13.9%, 5.8% and 6.7% respectively. High inflammatory status, defined by CRP and/or AGP, was observed in 16.2% of the children. Furthermore, the prevalence of DBM at individual level was assessed using various combinations of indicators such as individuals with concurrent undernutrition (those who were either stunted, underweight and/or wasted/thin, or solely stunted) or overnutrition (‘overweight’), in combination with each form of MND, as reported in online supplemental table S3. Overall, the co-existence of DBM at individual level was relatively low, ranging from 0.4% to 7.2%. When combining anaemia, ID, low serum retinol and low serum zinc, 58.5% of the children had at least one of four conditions, with 34.2% having one, 16.9% having two, 5.8% having three and 1.6% having four conditions.

Prevalence of different forms of malnutrition by area of residence and age group are illustrated in figure 1. Child stunting, underweight and wasting were more prevalent in mountainous areas compared with rural and urban areas. Prevalence of stunting was higher in children aged 2–4 years (15% vs 10.9%, and 11.0% in children aged <2 and 5–9 years, respectively), particularly those from the mountainous areas (34.1%). Underweight and wasting were more prevalent among older children aged 5–9 years (12.7% and 6.3%, respectively). Overweight and obesity were more common among older children aged 5–9 years (23.0%) compared with those aged <2 (3.0%) and 2–4 (5.3%) years and was particularly evident in children aged 5–9 years from the urban areas (31.4%).

Anaemia, ID, IDA and low serum retinol were more prevalent among younger children aged <2 years than in older children aged 2–4 and 5–9 years, particularly if they were from the mountainous areas as opposed to urban areas. Over two-fifths (43.7%) of the children aged <2 years from mountainous areas were affected by anaemia (vs 16.0% and 19.7% in children aged 2–4 and 5–9 years) and ID (47.5%) (vs 12.2% and 7.4% in children aged 2–4 and 5–9 years), and about one-third (29.0%) were affected by low serum retinol (vs 27.0% and 14.7% in children aged 2–4 and 5–9 years), whereas higher prevalence of low serum zinc was observed in older children aged 5–9 years, especially in children from mountainous areas (74.3%) compared with rural (48.3%) and urban (45.6%) areas.

### Effects of demographic and socioeconomic status on different forms of malnutrition

The associations between demographic and socioeconomic indicators and undernutrition or overnutrition, and MNDs are reported in tables2  3, respectively. Compared with children aged <2 years, children aged 2–4 years had higher odds of being stunted (OR (95% CI) 1.43 (1.20, 1.72)), while children aged 5–9 years had higher odds of being wasted (2.48 (1.49, 4.14)) (table 2). In addition, children aged 2–4 years had significantly increased odds of underweight (1.70 (1.29, 2.23)) and overweight (1.75 (1.14, 2.70)), with further increases in older children aged 5–9 years (2.46 (1.70, 3.56); 10.02 (6.71, 14.97), respectively). In contrast, compared with the youngest children, children aged 2–4 years had significantly reduced odds of anaemia (0.30 (0.25, 0.37)), low serum retinol (0.32 (0.25, 0.42)) and low serum zinc (0.82 (0.69, 0.97)) (table 3). The odds further decreased for children aged 5–9 years for anaemia (0.24 (0.19, 0.31)), ID (0.11 (0.07, 0.18)), IDA (0.11 (0.07, 0.18)) and low serum retinol (0.21 (0.15, 0.29)).

**Table 2:** Univariate logistic regressions between anthropometric indicators of malnutrition and demographic and socioeconomic indicators in Vietnamese children

Variables	Stunting	Underweight	Wasting/Thinness	Overweight and obesity
	OR (95% CI)	OR (95% CI)	OR (95% CI)	OR (95% CI)
Age (<2 years)	Ref	Ref	Ref	Ref
2–4 years	1.43 (1.20, 1.72)***	1.70 (1.29, 2.23)**	0.76 (0.49, 1.17)	1.75 (1.14, 2.70)*
5–9 years	1.10 (0.84, 1.45)	2.46 (1.70, 3.56)***	2.48 (1.49, 4.14)**	10.02 (6.71, 14.97) ***
Sex (females)	Ref	Ref	Ref	Ref
Males	1.03 (0.85, 1.25)	0.93 (0.76, 1.14)	0.90 (0.61, 1.33)	1.43 (1.16, 1.76)**
Area of residence (urban)	Ref	Ref	Ref	Ref
Rural	1.45 (0.86, 2.46)	1.32 (0.86, 2.02)	1.46 (0.90, 2.37)	0.71 (0.46, 1.10)
Mountainous	5.06 (1.81, 14.42)**	2.98 (1.50, 5.92)**	1.54 (0.59, 4.03)	0.25 (0.08, 0.77)*
Ethnicity (Kinh)	Ref	Ref	Ref	Ref
Others	5.94 (3.78, 9.36)***	3.68 (2.81, 4.82)***	1.56 (0.71, 3.44)	0.15 (0.07, 0.34)***
Geographical area (Northern mountains)	Ref	Ref	Ref	Ref
Red River Delta	0.29 (0.09, 0.96)*	0.44 (0.19, 0.99)*	1.01 (0.52,1.96)	1.87 (0.97, 3.60)
North Central and Central Coastal	0.50 (0.14, 1.75)	0.55 (0.21, 1.45)	0.86 (0.48, 1.56)	1.62 (0.86, 3.07)
Central Highlands	0.76 (0.17, 3.42)	1.09 (0.35, 3.36)	2.03 (0.74, 5.57)	1.38 (0.54, 3.50)
Southeast	0.19 (0.05, 0.74)*	0.21 (0.09, 0.47)**	0.71 (0.32, 1.60)	3.88 (2.07, 7.25)***
Mekong River Delta	0.28 (0.09, 0.91)*	0.65 (0.29, 1.49)	1.25 (0.75, 2.10)	2.90 (1.46, 5.79)**
Wealth index quintiles (poorest; Q1)	Ref	Ref	Ref	Ref
Poorer (Q2)	0.40 (0.19, 0.82)*	0.46 (0.31, 0.68)**	1.02 (0.38, 2.71)	2.94 (1.20, 7.21)*
Middle (Q3)	0.23 (0.11, 0.50)**	0.27 (0.19, 0.40)***	0.59 (0.23, 1.49)	2.56 (1.04, 6.29)*
Richer (Q4)	0.20 (0.09, 0.43)***	0.26 (0.16, 0.43)***	0.65 (0.25, 1.67)	2.65 (1.13, 6.21)*
Richest (Q5)	0.13 (0.05, 0.32)***	0.17 (0.10, 0.29)***	0.50 (0.20, 1.27)	2.91 (1.20, 7.05)*

OR and 95% CI were estimated using univariate logistic regression analyses. ‘Non-malnourished’ groups were used as the reference group.

*P<0.05; **p<0.01; ***p<0.001.

ref, reference.

**Table 3:** Univariate logistic regressions between micronutrient deficiencies and demographic and socioeconomic indicators in Vietnamese children

Variables	Anaemia	Iron deficiency	Iron deficiency anaemia	Low serum retinol	Low serum zinc
	OR (95% CI)	OR (95% CI)	OR (95% CI)	OR (95% CI)	OR (95% CI)
Age (<2 years)	Ref	Ref	Ref	Ref	Ref
2–4 years	0.30 (0.25, 0.37)***	0.26 (0.21, 0.32)	0.26 (0.21, 0.32)	0.32 (0.25, 0.42)***	0.82 (0.69, 0.97)*
5–9 years	0.24 (0.19, 0.31)***	0.11 (0.07, 0.18)***	0.11 (0.07, 0.18)***	0.21 (0.15, 0.29)***	0.71 (0.48, 1.08)
Sex (females)	Ref	Ref	Ref	Ref	Ref
Males	1.23 (1.02, 1.48)*	1.50 (1.22, 1.85)**	1.50 (1.22, 1.85)**	1.05 (0.83, 1.33)	1.11 (0.98, 1.25)
Area of residence (urban)	Ref	Ref	Ref	Ref	Ref
Rural	1.02 (0.70, 1.47)	1.01 (0.83, 1.22)	1.03 (0.80, 1.33)	1.07 (0.79, 1.47)	1.37 (0.92, 2.03)
Mountainous	2.07 (1.46, 2.94)***	1.09 (0.79, 1.51)	2.02 (1.32, 3.08)**	1.50 (0.88, 2.55)	2.29 (1.70, 3.09)***
Ethnicity (Kinh)	Ref	Ref	Ref	Ref	Ref
Others	2.13 (1.72, 2.64)***	2.07 (1.35, 3.17)**	2.07 (1.35, 3.17)**	2.34 (1.44, 3.79)**	1.93 (1.46, 2.56)***
Geographical area (Northern mountains)	Ref	Ref	Ref	Ref	Ref
Red River Delta	0.58 (0.43, 0.79)**	0.91 (0.67, 1.23)	0.51 (0.33, 0.80)**	0.87 (0.50, 1.51)	0.49 (0.26, 0.94)*
North Central and Central Coastal	0.56 (0.32, 0.98)*	0.99 (0.64, 1.53)	0.58 (0.33, 1.01)	1.20 (0.73, 1.95)	0.60 (0.33, 1.08)
Central Highlands	0.81 (0.56, 1.17)	1.11 (0.83, 1.48)	0.82 (0.54, 1.24)	1.025 (0.63, 1.66)	0.95 (0.63, 1.45)
Southeast	0.50 (0.37, 0.68)***	1.35 (0.99, 1.84)	0.66 (0.45, 0.98)*	1.17 (0.68, 2.02)	0.53 (0.30, 0.93)*
Mekong River Delta	0.55 (0.46, 0.67)***	1.23 (0.87, 1.73)	0.74 (0.46, 1.17)	1.435 (0.77, 2.66)	0.72 (0.43, 1.22)
Wealth index quintiles (poorest; Q1)	Ref	Ref	Ref	Ref	Ref
Poorer (Q2)	1.08 (0.75, 1.55)	1.12 (0.73, 1.73)	1.12 (0.73, 1.73)	0.92 (0.52, 1.63)	0.77 (0.45, 1.31)
Middle (Q3)	0.63 (0.44, 0.90)*	0.69 (0.43, 1.10)	0.69 (0.43, 1.10)	0.71 (0.42, 1.20)	0.54 (0.37, 0.78)**
Richer (Q4)	0.75 (0.48, 1.19)	0.67 (0.41, 1.10)	0.67 (0.41, 1.10)	0.64 (0.36, 1.12)	0.67 (0.45, 0.98)*
Richest (Q5)	0.55 (0.40, 0.76)**	0.46 (0.28, 0.77)**	0.46 (0.28, 0.77)**	0.41 (0.24, 0.72)**	0.43 (0.30, 0.64)***

OR and 95% CI were estimated using univariate logistic regression analyses. ‘Micronutrient non-deficient’ groups were used as the reference group.

*P<0.05; **p<0.01; ***p<0.001.

ref, reference.

Males were more likely to be overweight (OR (95% CI) 1.43 (1.16, 1.76)) and affected by anaemia (1.23 (1.02, 1.48)), ID (1.50 (1.22, 1.85)) and IDA (1.50 (1.22, 1.85)) compared with females (tables2  3). Children from mountainous areas had increased odds of stunting (5.06 (1.81, 14.42)), underweight (2.98 (1.50, 5.92)), low serum zinc (2.29 (1.70, 3.09)) and anaemia (2.07 (1.46, 2.94)) compared with children from urban areas. Ethnic minorities had increased odds of stunting (5.94 (3.78, 9.36)), underweight (3.68 (2.81, 4.82)) and all MNDs investigated (OR ranging from 1.93 to 2.34), but reduced odds of overweight (0.15 (0.07, 0.34)). The odds of being stunted and underweight were significantly decreased across wealth index quintiles, with children from the richest quintile (Q5) most protected against stunting (0.13 (0.05, 0.32)), and underweight (0.17 (0.10, 0.29)), compared with the poorest quintile (Q1) (table 2). In contrast, children from greater wealth index quintiles (Q2–Q5) had significantly increased odds of overweight (OR ranging from 2.6 to 2.9). In addition, children from the richest quintile (Q5) were also protected against anaemia (0.55 (0.40, 0.76)), ID (0.46 (0.28, 0.77)) and IDA (0.46 (0.28, 0.77)), low serum retinol (0.41 (0.24, 0.72)) and low serum zinc (0.43 (0.30, 0.64)) (table 3).

Compared with the Northern mountains, children from the Southeast and Mekong River Delta had reduced odds of stunting (OR (95% CI) 0.19 (0.05, 0.74) and 0.28 (0.09, 0.91)), but had increased odds of overweight (3.88 (2.07, 7.25) and 2.90 (1.46, 5.79)), respectively (table 2). Children from the Red River Delta and the Southeast had also reduced odds of underweight (0.44 (0.19, 0.99) and 0.21 (0.09, 0.47), respectively). Moreover, children from the Red River Delta and the Southeast were less likely to be affected by anaemia, IDA and low serum zinc (OR ranging from 0.49 to 0.66) (table 3).

### Associations between anthropometric indicators of malnutrition (undernutrition and overnutrition) and micronutrient deficiencies

In the adjusted multivariate logistic regressions, children with anaemia (AOR (95% CI) 1.43 (1.13, 1.82)), IDA (1.53 (1.22, 1.93)) and low serum zinc (1.44 (1.11, 1.88)) had significantly increased odds of stunting compared with the non-deficient children (table 4). Children with low serum retinol (1.71 (1.21, 2.42)) and low serum zinc (1.51 (1.13, 2.01)) had increased odds of being underweight, and children with low serum retinol also had reduced odds of being overweight (0.43 (0.27, 0.68)). When combining the number of MNDs (including anaemia) occurring in each child, we found that children with three MNDs (1.96 (1.14, 3.37)) and children with four MNDs (1.84 (1.08, 3.13)) had increased odds of stunting, compared with those without MNDs. Similarly, children with two, three and four MNDs had increased odds of being underweight in a linear manner (1.48 (1.01, 2.17), 1.81 (1.17, 2.81) and 2.26 (1.17, 4.35), respectively). Children with one, two and three MNDs were less likely to be overweight or obese (0.60 (0.44, 0.81), 0.38 (0.23, 0.63) and 0.44 (0.23, 0.85), respectively).

**Table 4:** Univariate and multivariate logistic regressions between anthropometric indicators of malnutrition (undernutrition and overnutrition) and micronutrient deficiencies in Vietnamese children

Variables	Stunting		Underweight		Wasting/Thinness		Overweight and obesity	
	OR (95% CI)	AOR (95% CI)	OR (95% CI)	AOR (95% CI)	OR (95% CI)	AOR (95% CI)	OR (95% CI)	AOR (95% CI)
Anaemia (no)†	Ref	Ref	Ref	Ref	Ref	Ref	Ref	Ref
Yes	1.39 (1.14, 1.70)**	1.43 (1.13, 1.82)**	1.09 (0.84, 1.42)	1.33 (0.99, 1.78)	0.79 (0.51, 1.24)	0.88 (0.53, 1.47)	0.57 (0.38, 0.85)**	0.76 (0.50, 1.15)
Iron deficiency (no)‡	Ref	Ref	Ref	Ref	Ref	Ref	Ref	Ref
Yes	1.17 (0.90, 1.52)	1.11 (0.88, 1.40)	0.81 (0.52, 1.25)	1.01 (0.68, 1.51)	0.49 (0.25, 0.95)*	0.65 (0.33, 1.26)	0.63 (0.50, 0.79)**	1.07 (0.79, 1.44)
Iron deficiency anaemia (no)‡	Ref	Ref	Ref	Ref	Ref	Ref	Ref	Ref
Yes	1.52 (1.05, 2.18)*	1.53 (1.22, 1.93)*	1.00 (0.60, 1.67)	1.33 (0.76, 2.24)	0.44 (0.25, 0.76)**	0.56 (0.32, 1.01)	0.54 (0.35, 0.85)*	1.03 (0.58, 1.84)
Low serum retinol (no)†	Ref	Ref	Ref	Ref	Ref	Ref	Ref	Ref
Yes	1.55 (1.02, 2.36)*	1.40 (0.86, 2.30)	1.47 (1.04, 2.07)*	1.71 (1.21, 2.42)**	1.02 (0.60, 1.72)	1.21 (0.73, 1.99)	0.34 (0.20, 0.57)***	0.43 (0.27, 0.68)***
Low serum zinc (no)†	Ref	Ref	Ref	Ref	Ref	Ref	Ref	Ref
Yes	1.49 (1.12, 1.97)**	1.44 (1.11, 1.88)**	1.43 (1.07, 1.91)*	1.51 (1.13, 2.01)**	1.15 (0.71, 1.87)	1.08 (0.66, 1.76)	0.63 (0.37, 1.05)	0.65 (0.38, 1.10)
Total number of MND (none)†	Ref	Ref	Ref	Ref	Ref	Ref	Ref	Ref
1	1.02 (0.74, 1.41)	1.05 (0.72, 1.51)	0.88 (0.68, 1.15)	0.98 (0.72, 1.34)	0.71 (0.42, 1.20)	0.87 (0.53, 1.44)	0.48 (0.36, 0.63)***	0.60 (0.44, 0.81)**
2	1.51 (1.07, 2.14)*	1.58 (0.99, 2.52)	1.05 (0.76, 1.46)	1.48 (1.01, 2.17)*	0.69 (0.40, 1.21)	1.01 (0.57, 1.78)	0.22 (0.15, 0.31)***	0.38 (0.23, 0.63)**
3	1.67 (1.17, 2.40)**	1.96 (1.14, 3.37)*	0.95 (0.58, 1.54)	1.81 (1.17, 2.81)*	0.59 (0.29, 1.22)	0.92 (0.48, 1.77)	0.16 (0.09, 0.29)***	0.44 (0.23, 0.85)*
4	1.58 (1.04, 2.40)*	1.84 (1.08, 3.13)*	1.05 (0.59, 1.89)	2.26 (1.17, 4.35)*	0.30 (0.07, 1.34)	0.49 (0.12, 2.06)	0.31 (0.12, 0.79)*	1.12 (0.43, 2.92)

Univariate and multivariate logistic regression analyses were performed and reported as OR (95% CI) and AOR (95% CI), respectively. ‘Micronutrient non-deficient’ groups were used as the reference group.

*P<0.05; **p<0.01; ***p<0.001.

†Adjusted by age, sex, area of residence and inflammation.

‡Adjusted by age, sex and area of residence.

AOR, adjusted OR; MND, micronutrient deficiencies; ref, reference.

## Discussion

Our findings indicate that the DBM remains a major public health issue for Vietnamese children. This survey shows that the prevalence of stunting in children aged <2 and 2–4 years was 10.9% and 15.0%, respectively, in 2020. These results suggest that Vietnam is now on track to achieve the Global Nutrition Target of reducing stunting in children under 5 years by 40% by 2025, compared with 2012, when the prevalence was <20%.^7 27^ In contrast, Vietnam is off track to meet the 2025 target for childhood overweight and obesity, which is particularly evident among school-aged children in urban areas (31%). This prevalence has substantially increased compared with 8.5% in 2010for children of same age.^12^ MNDs, particularly low serum zinc, anaemia and ID, were widespread among children in Vietnam, although low serum retinol was less of a concern (<7%). In line with previous findings on Vietnamese children across various age groups, this survey reveals that more than half of children aged 6 months to 9 years had low serum zinc status.^10 28^ Anaemia and ID affected 15% and 13% of participants, respectively. Males were disproportionately affected by overweight and MNDs, including anaemia, ID and IDA.

Our findings demonstrated significant demographic and socioeconomic inequalities in child malnutrition in Vietnam. Consistent with previous findings in Vietnam, our results underscored that stunting was more prevalent among children aged 2–4 years, while underweight, wasting and overweight were more common among older children aged 5–9 years.^10^ All the MNDs investigated were more frequently observed in younger children aged 6–23 months. This aligns with findings from studies of feeding practice in children of this age, which reveal a high prevalence of suboptimal complementary feeding practices and poor dietary quality of complementary foods in Vietnam, such that only 36.7% of children achieved minimum dietary diversity and 29.0% achieved minimum acceptable diet, contributing to nutritional deficiencies.^29^

Vietnamese diets have shifted from a reliance on starchy staples, dark-green vegetables and legumes to an increased consumption of animal proteins, fats/oils, processed foods and beverages.^30^ Despite a decreasing demand for starchy staples such as rice and grains, these foods remain the primary source of calories in the Vietnamese diet.^31^ Rice is low in zinc and high in phytic acid, which binds to zinc and forms phytate salts, inhibiting zinc and iron absorption, which could potentially contribute to the development of deficiencies.^32^ Although increased consumption of meat, egg and dairy products can contribute to essential micronutrient intake especially for iron, zinc, folate and calcium,^32 33^ the rising intake of micronutrient-poor processed foods and sugary beverages may negate these health benefits. Unhealthy diets, particularly the consumption of sugary sweetened beverages and fast foods, along with physical inactivity, poor sleep, maternal obesity, gestational diabetes during pregnancy and suboptimal feeding practices, are the major risk factors for overweight and obesity in Vietnamese school-aged children and adolescents, especially in developed areas.^1234^,^36^

This research found that stunting, underweight and MNDs (including anaemia, IDA and low serum zinc) were more prevalent among children from ethnic minorities, poorer families and those living in rural or mountainous areas of the Northern and Central Highlands. Conversely, overweight and obesity were predominantly observed in wealthier families and urban areas, particularly in the Mekong River Delta and Southeast regions. Previous evidence reported that ethnic minorities living in poor and remote areas had limited economic, social or cultural support including education and health services compared with the major ethnic groups (Kinh or Hoa).^13 37^ A recent study also reported that nearly 70% of low-income households, particularly in the Northern midlands and mountainous areas, struggle to afford a healthy diet, as defined by food-based dietary guidelines.^31^ Additionally, rural households had lower dietary diversity scores compared with urban households, primarily due to a greater reliance on home-produced foods such as rice and legumes, as well as limited nutritional knowledge and reduced income, which leads to restricted purchasing power.^38^ Together, these factors contribute to persistent disparities in nutritional outcomes, resulting in a higher prevalence of undernutrition and MNDs in disadvantaged areas.^39^ Targeted efforts are required to reduce the socioeconomic inequality gap and improve nutrition among ethnic minorities and vulnerable populations in remote, impoverished regions.^40^

Caution should be exercised when assessing and interpreting zinc deficiency using plasma or serum samples, due to various influencing factors, including the timing of blood collection, meal consumption, fasting or non-fasting status and inflammation.^41^ In our study, although non-fasting blood samples were collected between 07:00 and 09:00 hours to minimise variability related to diurnal fluctuations in serum zinc levels, potential contamination of blood samples may underestimate or overestimate the prevalence of zinc deficiency. To date, there is no consensus on the best indicators to assess zinc status, making it difficult to compare and assess the extent of zinc deficiency in a population.^41^ Nonetheless, plasma or serum zinc remains a frequently used biomarker at population level.^42^ Effective public health solutions may include zinc supplementation^43^ or bio-fortification of zinc rich foods, for example, maize, wheat or rice.^44^

Low serum retinol can lead to impaired immune system function, increasing the risk of infectious diseases and child morbidity and mortality.^45^ In 2012, Laillou *et al* reported that 11.8% of children aged <2 years and 11.9% of children aged 2–5 years had low serum retinol.^28^ Concerningly, within a decade, the prevalence of low serum retinol has increased substantially, with 22.5% children aged <2 years and 18.0% of children aged 2–5 years in this study found to have low serum retinol. This increase may be influenced by urban-rural difference, as our study had a larger proportion of rural children (77.6% vs 48.4%). Notably, Vietnam has stipulated mandatory vitamin A fortification in sugar and vegetable oil^46^ and implemented vitamin A supplementation programme targeted at children aged 6–36 months since 1997.^47^ However, the coverage of vitamin A supplementation varies across regions, with 70%–80% in most regions, but significant lower rates (~60%) in certain remote regions, for example, Central Highlands and North-West regions. This discrepancy may explain the higher prevalence of low serum retinol in mountainous areas (14.7%–29.0%) compared with urban settings (<10%). These findings underscore the needs to reassess the current design of vitamin A supplementation and fortification programmes, to ensure they effectively reach the most vulnerable populations. Consumption of vitamin A-rich fruits and vegetables should also be encouraged to sustainably combat vitamin A deficiency.

Higher prevalence of anaemia in children aged <2 years has been reported in many other populations,^48 49^ but not all.^50^ In the first 4–6 months of life, iron supply acquired in utero is sufficient to meet requirements of infants with unimpaired intrauterine supply, whereas after 6 months, the intrauterine-supplied iron becomes insufficient due to accelerated growth and development, leading to increased risk of deficiency.^51^ Due to immaturity of gut microbiome and immune system, infants and young children are more susceptible to infections and diseases, which increases the risk of nutritional deficiencies.^52^ Increased risk of anaemia in males during early childhood compared with females has also been reported in many studies.^53^,^56^ Previous studies reported significantly lower cord serum ferritin levels and higher cord sTfR in males than in females at birth and during first 2 years of life, which suggests that males were at greater risk of low iron status.^57^ Although the underlying mechanisms are unknown, this may be explained by hormone-mediated differences in metabolism for example, increased erythropoietin activity for red blood cell production in males may result in low iron store.^58^ Differences in the proportion of lean and fat mass and weight gain (eg, greater weight gain in males) may also indirectly affect iron metabolism.^59^

ID is frequently reported as the main nutritional contributor to anaemia, but the aetiology of anaemia is complex, multifactorial and context-specific.^60^ Our findings indicate that nearly one-third of the anaemia cases in Vietnamese children were attributable to ID. The proportion of anaemia attributable to ID was highest in children aged <2 years (57%) compared with older children aged 5–9 years (24.4%). This finding was in line with previous evidence, which reported that the prevalence of anaemia attributable to ID was highest in the 1–4 years age group, and then gradually decreased up to the 25–29 years age group.^54^ Caution should be taken when interpretating the results, as unmeasured factors such as a high burden of disease, infection or haemoglobinopathies may overestimate the proportion of anaemia attributable to ID.^61^ Other important known risk factors of childhood anaemia include nutritional deficiencies (eg, iron, vitamin A, folate, vitamin B_12_), environmental factors, chronic comorbidities (eg, infection, intestinal parasites, malaria) and congenital or genetic disorders (eg, sickle cell, α-thalassemia), which are further complicated by socioeconomic and ecological risk factors.^62^ Therefore, these risk factors should be considered when investigating the underlying causes of anaemia, in order to develop appropriate and effective integrated interventions to prevent and treat childhood anaemia in Vietnam.

Our findings showed that MNDs were highly associated with child stunting and underweight. Inadequate intake of micronutrients, especially iron, zinc, retinol and vitamin C may result in growth retardation.^63^ Low energy and poor nutrient diets are acknowledged as the main cause of both stunting and MNDs, other factors include those associated with poverty or poor socioeconomic development such as poor sanitation and hygiene, infection, poor maternal nutrition and feeding practices.^64^ Despite excessive energy consumption, previous evidence demonstrated that children living with overweight and obesity were more likely to be affected by MNDs, and this may be primarily due to energy-dense nutrient-poor diets that are low in micronutrient content.^65^ Our study found contradictory results, suggesting that overweight may protect against the development of multiple forms of MNDs. Possible explanations include that overweight children and children with obesity may consume more food, which enables them to meet their nutritional requirements, or that fortification of certain processed foods may be more accessible and thus more likely to be consumed by these children.^66^ To date, the direction of the association between overnutrition and MNDs is still unknown.^67^ It is unclear whether specific MNDs contribute to increasing risk of greater obesity and adiposity, or whether increased overweight and adiposity resulted in specific MNDs, or both. The link between overnutrition and MNDs is further complicated by the nutrient density as diets containing similar amounts of energy can vary in terms of the range of micronutrients that they provide.^68^ Thus, future research is warranted to shed light on the relationship between overnutrition and MNDs.

### Policy implications

National policies and intervention programmes should address age-specific, sex-specific, geographical and socioeconomic disparities to ensure that any programme reaches those at greatest risk and in greatest need. Undernutrition and MNDs were more prevalent among younger children aged <2 years in this study, highlighting the importance of optimising nutrition in early life to alleviate undernutrition and MNDs in Vietnamese children. Adequate maternal and early life nutrition, especially during the first 1000 days of life, is essential to ensure optimal growth and ensure children develop to their full potential.^69^ Thus, interventions such as multimicronutrient supplementation and food fortification during pregnancy and infancy should be targeted during this critical period. Nutrition education to optimise infant and young children feeding practices (eg, breastfeeding and complementary feeding), and to promote dietary diversification using locally available micronutrient-rich foods may help to tackle multiple forms of malnutrition in infant and young children.^70^

In recent years, the Vietnamese government has implemented the National Nutrition Strategy 2021–2030, with a vision extending to 2040, which includes targets and indicators related to childhood overweight and obesity.^12^ However, current policies and guidelines in Vietnam were not specifically designed to address childhood overweight and obesity, rather all the programmes were integrated or designed as a small part of broader public health strategies.^71^ Such policies and guidelines included taxes on sugary-sweetened beverages, nutrition labelling, restriction on marketing unhealthy foods on school campuses, physical activity programmes and national dietary guidelines. Further obesity prevention research is needed to provide sufficient evidence to inform policy decisions aimed at addressing childhood overweight and obesity. It is also essential to raise public awareness about the consequences of and risk factors for overweight and obesity to generate demands for healthier options, strengthen nutrition education and promote healthy eating behaviours and lifestyles in both children and their parents/caregivers.

### Strengths and limitations

This study had some notable strengths. The nationally representative sample of Vietnamese children allows the findings to be generalised across the country. Stratified analyses by age groups, area of residence and sex provide insights into the vulnerable population in Vietnam who are at the greatest risk of developing multiple forms of malnutrition. These findings can help to better tailor interventions, ensuring they reach those who most need to improve outcomes. There were some limitations of this study. As in all observational studies, this study is subjected to confounding, and causality should not be inferred. We acknowledged that the exclusion of other micronutrients such as folate, iodine, vitamin B_12_ and vitamin D is a shortcoming, primarily due to funding and resource constraints. We recommend analysing these additional micronutrients in future studies to provide additional comprehensive understanding of malnutrition in the Vietnamese population. Food consumption data in children was not available in the GNS 2020, thus analysis of the association between food intake and child nutritional status was not possible.

## Conclusion

Our findings indicate that the DBM is a major public health concern for Vietnamese children. Demographic variation and socioeconomic inequalities in DBM remain critical challenge for policymakers. Undernutrition and MNDs are more prevalent in infants and young children, particularly those from ethnic minorities, rural and mountainous areas and poor families. Conversely, the increasing prevalence of childhood overweight and obesity is evident, particularly in males, school-aged children from urban areas and richer families. Thus, national policies and intervention programmes in Vietnam should address age-specific, sex-specific, geographical and socioeconomic disparities to ensure equitable access and benefits for all. This is crucial to accelerate progress in improving child nutrition. Existing food and nutrition programmes and policies need to be redesigned to use a multisectoral, double-duty approach to simultaneously address all forms of child malnutrition.

## Supplementary material

10.1136/bmjph-2024-001177 online supplemental file 1

## Data Availability

Data are available on reasonable request.
