# Supplementary material for: Demographic variation and socioeconomic inequalities in all forms of malnutrition among children aged 6 months to 9 years: findings from the Vietnamese General Nutrition Survey 2020
Source: BMJ Public Health. 2025 Feb 4;3(1):e001177. doi: 10.1136/bmjph-2024-001177 (PMC12320070; doi:10.1136/bmjph-2024-001177)
Supplement: online supplemental file 1 [file bmjph-3-1-s001.pdf]

Demographic variation and socioeconomic inequalities in all forms of malnutrition among children aged 6 months to 9 years: Findings from the Vietnamese General Nutrition Survey 2020

P.Y. Tan[1], S. V. Som[1,2], S. D. Nguyen[3,4], X. Tan[1], D.T. Tran[4], N.T. Tran[5], V.K. Tran[5], L. Dye[1,6], J. B. Moore[1], S. Caton[7], H. Ensaff[1], X. Lin[8], G. Smith[9], Y. Y. Gong[1]

Supplementary materials

Supplementary Table S1: Number of observations and missing data for each variable

Variable	Number of observations	Missing data (%)
**Demographic and socioeconomic indicators**		
Age	7,829	0
Sex	7,829	0
Area of residence	7,829	0
Geographical area	7,829	0
Ethnicity	7,829	0
Wealth index	7,829	0
**Anthropometric indicators of malnutrition**		
Stunting	6,796	13.2
Underweight	6,813	13.0
Wasting/thinness	6,790	13.3
Overweight	6,790	13.3
**Biomarkers**		
Anaemia	7,249	7.4
Iron deficiency	6,800	13.1
Iron deficiency anaemia	7,249	7.4
Low serum retinol	7,124	9.0
Low serum zinc	5,271	32.7
Inflammation (CRP and AGP)	6,831	12.7

AGP, α1-acid glycoprotein; CRP, c-reactive protein.

Supplementary Table S2: General characteristics, anthropometric parameters, and micronutrient biomarkers of the Vietnamese children by anthropometric indicators of malnutrition

Variables	Total	Non-stunted	Stunted	p value	Non-underweight	Underweight	p value	Non-wasted	Wasted	p value	Non-overweight	Overweight	p value
**Demographic and socioeconomic indicators**													
Age (years)	5 ± 3	5 ± 3	5 ± 3		5 ± 3	6 ± 3	***	5 ± 3	6 ± 3	**	5 ± 3	7 ± 2	***
Age group													
<2 years	2039 (16.0)	1521 (15.8)	213 (13.3)	**	1622 (16.3)	117 (8.2)	***	1669 (15.6)	64 (11.1)	***	1681 (17.4)	52 (3.2)	***
2-4 years	3513 (31.6)	2602 (30.4)	446 (36.7)		2783 (31.6)	272 (27.1)		2951 (31.7)	83 (16.6)		2855 (34.3)	179 (11.3)	
5-9 years	2277 (52.4)	1794 (53.8)	220 (50.0)		1766 (52.1)	253 (64.7)		1895 (52.7)	128 (72.3)		1565 (48.2)	458 (85.5)	
Sex													
Males	3958 (51.5)	2935 (48.3)	423 (47.5)		3048 (47.8)	315 (49.6)		3220 (48)	142 (52.1)		3064 (49.5)	298 (40.5)	**
Females	3871 (48.5)	2982 (51.7)	456 (52.5)		3123 (52.2)	327 (50.4)		3295 (52)	133 (47.9)		3037 (50.5)	391 (59.5)	
Ecological area													
Northern mountains	1207 (15.5)	807 (13.9)	296 (33.8)	*	960 (15.3)	155 (26.4)	*	1067 (16.3)	38 (17.4)		1053 (17.9)	52 (7.1)	**
Red River Delta	1395 (26.6)	1187 (29.1)	111 (19.5)		1199 (28.7)	103 (21.7)		1244 (28.1)	60 (27.2)		1177 (28.0)	127 (28.7)	
North Central and Central Coastal	1466 (22.4)	1147 (22.4)	159 (24.8)		1202 (22.8)	106 (21.7)		1261 (22.7)	46 (21.9)		1179 (23.4)	128 (18.6)	
Central Highlands	1157 (6.1)	713 (4.8)	156 (7.9)		732 (4.7)	137 (8.9)		812 (5.0)	55 (7.7)		813 (5.4)	54 (3.4)	
Southeast	1218 (15.2)	941 (14.6)	55 (4.8)		955 (14.1)	36 (5.1)		961 (13.4)	24 (9.4)		833 (11.7)	152 (22.1)	
Mekong River Delta	1386 (14.3)	1122 (15.3)	102 (9.2)		1123 (14.3)	105 (16.2)		1170 (14.4)	52 (16.3)		1046 (13.5)	176 (20)	
Area of residence													
Urban	2540 (30.1)	2005 (31.2)	190 (17.4)	*	2020 (30.4)	175 (20.7)	*	2103 (29.8)	86 (22.3)		1928 (27.8)	261 (39.2)	*
Rural	5289 (69.9)	3912 (68.8)	689 (82.6)		4151 (69.6)	467 (79.3)		4412 (70.2)	189 (77.7)		4173 (72.2)	428 (60.8)	
Ethnicity													
Kinh	6419 (85.0)	5160 (89.3)	494 (58.5)	***	5260 (87.7)	406 (66.0)	***	5441 (85.9)	208 (76.9)		4985 (83.5)	664 (97.1)	***
Others	1410 (15.0)	757 (10.7)	385 (41.5)		911 (12.3)	236 (34.0)		1074 (14.1)	67 (23.1)		1116 (16.5)	25 (2.9)	
Wealth index													
Poorest (Q1)	1074 (13.6)	632 (10.6)	294 (34.5)	***	738 (11.7)	196 (32.2)	***	878 (13.5)	53 (20.8)		895 (15.2)	36 (6.0)	*
Poorer (Q2)	1735 (16.3)	1258 (15.2)	228 (19.5)		1327 (15.3)	161 (19.1)		1407 (15.4)	73 (21.3)		1320 (15.3)	160 (17.8)	
Middle (Q3)	2029 (22.6)	1536 (22.9)	150 (17.1)		1571 (22.7)	118 (17.1)		1634 (22.3)	54 (18.4)		1507 (22.0)	181 (23.0)	
Richer (Q4)	1981 (30.2)	1599 (31.3)	151 (20.4)		1630 (30.7)	122 (22.3)		1680 (29.9)	63 (26.8)		1551 (29.4)	192 (31.7)	
Richest (Q5)	1010 (17.3)	892 (20.0)	56 (8.6)		905 (19.6)	45 (9.3)		916 (18.8)	32 (12.8)		828 (18.1)	120 (21.6)	
**Anthropometric parameters**													

Weight (kg)	19 ± 12.5	19 ± 8.9	14.5 ± 6	***	18.9 ± 8.4	14.3 ± 3.7	***	18.6 ± 8.5	15.5 ± 4.4	***	16.3 ± 5.8	31 ± 8.7	***
Height (cm)	105.5 ± 20.1	106.2 ± 19.2	96.1 ± 15.3	***	105.4 ± 19.9	100.9 ± 15.1	***	104.9 ± 19.6	109.5 ± 16.6	*	102.2 ± 18.8	121.9 ± 14.5	***
HAZ	-0.65 ± 1.28	-0.35 ± 1.05	-2.72 ± 0.70	***	-0.45 ± 1.16	-2.39 ± 0.96	***	-0.64 ± 1.28	-1.07 ± 1.13	***	-0.81 ± 1.24	0.30 ± 1.05	***
WAZ	-0.40 ± 1.42	-0.18 ± 1.28	-2.03 ± 0.89	***	-0.14 ± 1.25	-2.65 ± 0.59	***	-0.32 ± 1.37	-2.25 ± 0.86	***	-0.77 ± 1.11	1.75 ± 0.95	***
WHZ	-0.08 ± 1.14	-0.04 ± 1.15	-0.4 ± 0.99	***	0.03 ± 1.08	-1.41 ± 0.86	***	-0.01 ± 1.07	-2.55 ± 0.52	***	-0.23 ± 0.95	2.68 ± 0.78	***
BAZ	0.01 ± 1.34	0.03 ± 1.33	-0.33 ± 1.07	***	0.15 ± 1.25	-1.42 ± 0.98	***	0.11 ± 1.23	-2.53 ± 0.54	***	-0.39 ± 1.01	2.21 ± 0.69	***
BMI	16.3 ± 4.0	16.3 ± 3.9	15.3 ± 4.2	**	16.3 ± 2.8	13.3 ± 1.0	***	16.3 ± 2.7	12.6 ± 0.7	***	14.9 ± 1.4	20.3 ± 2.3	***
**Blood biomarkers**													
Haemoglobin (g/dL)	12.3 ± 1.2	12.3 ± 1.2	12.1 ± 1.3		12.3 ± 1.2	12.3 ± 1.1		12.3 ± 1.2	12.4 ± 1		12.2 ± 1.2	12.6 ± 1	***
Serum ferritin (μg/L)	39.7 ± 1.3	40.5 ± 1.4	38.4 ± 2.8		39.5 ± 1.4	45.5 ± 2.7	*	39.5 ± 1.4	52.9 ± 4.7	**	38.2 ± 1.4	54.2 ± 2.0	***
sTfR (mg/L)	6.2 ± 2.6	6.1 ± 2.4	6.5 ± 3.3	*	6.2 ± 2.6	6 ± 2.5		6.2 ± 2.6	5.7 ± 1.8	**	6.2 ± 2.7	5.9 ± 1.7	**
Body iron store	5.5 ± 3.9	5.6 ± 3.8	5.3 ± 4.1		5.5 ± 3.9	6.1 ± 3.5	*	5.5 ± 3.9	6.7 ± 2.8	**	5.3 ± 4.0	6.8 ± 2.9	***
Serum zinc (μmol/L)	9.9 ± 6.3	10 ± 6.3	8.9 ± 5.2	**	9.9 ± 6.2	9.1 ± 5.6	*	9.9 ± 6.2	8.9 ± 4.4	*	9.8 ± 6.1	10.8 ± 6.7	
Serum retinol (μmol/L)	1.2 ± 0.4	1.2 ± 0.4	1.1 ± 0.4		1.2 ± 0.4	1.1 ± 0.4		1.2 ± 0.4	1.1 ± 0.4		1.1 ± 0.4	1.3 ± 0.3	***
RBP (μmol/L)	1.1 ± 0.4	1.1 ± 0.4	1.1 ± 0.4		1.1 ± 0.4	1.1 ± 0.4		1.1 ± 0.4	1.1 ± 0.5		1.1 ± 0.4	1.3 ± 0.3	***
CRP (mg/L)	1.6 ± 4.5	1.6 ± 4.3	1.9 ± 5.0		1.6 ± 4.3	1.7 ± 4.4		1.6 ± 4.3	1.5 ± 4.0		1.1 ± 0.4	1.2 ± 0.4	***
AGP (mg/L)	0.8 ± 0.3	0.8 ± 0.3	0.8 ± 0.4		0.8 ± 0.3	0.8 ± 0.3		0.8 ± 0.3	0.8 ± 0.3		1.5 ± 4.3	2.2 ± 4.2	**

AGP, α-1-acid glycoprotein; CRP, C-reactive protein; RBP, Retinol-binding protein; sTfR, Serum transferrin receptor.
Continuous and categorical variables were reported as mean ± SD, and number (percentage), respectively.
P-value was based on t-test and Chi Square test for association for continuous and categorical variables, respectively. * $p<0.05$; ** $p<0.01$; *** $p<0.001$.
Concentration of serum ferritin (μg/L) was presented as geometric mean and standard error (SE).

Supplementary Table S3: Prevalence of individual-level double burden of malnutrition which assessed using various combinations of indicators

Indicators used	Prevalence (%)
Having multiple micronutrient deficiencies (MNDs) including anaemia, iron deficiency, low serum retinol, and low serum zinc)	
None	41.5
1 MND	34.2
2 MNDs	16.9
3 MNDs	5.8
4 MNDs	1.6
Concurrent stunting and overweight	0.4
Concurrent stunting and anaemia	1.8
Concurrent stunting and iron deficiency	1.4
Concurrent stunting and low serum retinol	5.1
Concurrent stunting and low serum zinc	3.5
Concurrent undernutrition (stunting, underweight or wasting/thinness) and anaemia	2.4
Concurrent undernutrition (stunting, underweight or wasting/thinness) and iron deficiency	1.8
Concurrent undernutrition (stunting, underweight or wasting/thinness) and low serum retinol	7.2
Concurrent undernutrition (stunting, underweight or wasting/thinness) and low serum zinc	4.4
Concurrent overweight and anaemia	1.0
Concurrent overweight and iron deficiency	1.1
Concurrent overweight and low serum retinol	3.4
Concurrent overweight and low serum zinc	0.9
